# Mobilization of Endogenous Bone Marrow Derived Endothelial Progenitor Cells and Therapeutic Potential of Parathyroid Hormone after Ischemic Stroke in Mice

**DOI:** 10.1371/journal.pone.0087284

**Published:** 2014-02-04

**Authors:** Li-Li Wang, Dongdong Chen, Jinhwan Lee, Xiaohuan Gu, Ghina Alaaeddine, Jimei Li, Ling Wei, Shan Ping Yu

**Affiliations:** 1 Department of Anesthesiology, Emory University School of Medicine, Atlanta, Georgia, United States of America; 2 Department of Neurology, Beijing Friendship Hospital, Capital Medical University, Beijing, China; 3 Department of Neurology, Emory University School of Medicine, Atlanta, Georgia, United States of America; William Harvey Research Institute, Barts and The London School of Medicine and Dentistry, Queen Mary University of London, United Kingdom

## Abstract

Stroke is a major neurovascular disorder threatening human life and health. Very limited clinical treatments are currently available for stroke patients. Stem cell transplantation has shown promising potential as a regenerative treatment after ischemic stroke. The present investigation explores a new concept of mobilizing endogenous stem cells/progenitor cells from the bone marrow using a parathyroid hormone (PTH) therapy after ischemic stroke in adult mice. PTH 1-34 (80 µg/kg, i.p.) was administered 1 hour after focal ischemia and then daily for 6 consecutive days. After 6 days of PTH treatment, there was a significant increase in bone marrow derived CD-34/Fetal liver kinase-1 (Flk-1) positive endothelial progenitor cells (EPCs) in the peripheral blood. PTH treatment significantly increased the expression of trophic/regenerative factors including VEGF, SDF-1, BDNF and Tie-1 in the brain peri-infarct region. Angiogenesis, assessed by co-labeled Glut-1 and BrdU vessels, was significantly increased in PTH-treated ischemic brain compared to vehicle controls. PTH treatment also promoted neuroblast migration from the subventricular zone (SVZ) and increased the number of newly formed neurons in the peri-infarct cortex. PTH-treated mice showed significantly better sensorimotor functional recovery compared to stroke controls. Our data suggests that PTH therapy improves endogenous repair mechanisms after ischemic stroke with functional benefits. Mobilizing endogenous bone marrow-derived stem cells/progenitor cells using PTH and other mobilizers appears an effective and feasible regenerative treatment after ischemic stroke.

## Introduction

According to recent statistics, stroke is the fourth leading cause of human death and the number one cause of disability in the adult population in the United States [1]. Moreover, approximately 5.7 million people each year die from stroke worldwide [2]. Among all stroke patients, 87% suffer from ischemic stroke [1]. Despite the economic, healthcare and social burden of stroke, stroke treatment is still limited to thrombolytic therapy using tissue plasminogen activator (tPA) with a narrow time window of 4.5 hrs after the onset of ischemic attack. For stroke survivors, during the sub-acute and chronic phases, only supportive care and rehabilitation are available with uncertain partial recovery. In short, stroke represents a clinical entity that requires more innovative treatments both for acute neuroprotection and for regenerative tissue repair.

Transplantation of exogenous stem cells and/or neural progenitor cells has shown promising results in the treatment of ischemic stroke in animal models and in clinical trials [3], [4], yet cell transplantation therapy continues to face both ethical and clinical issues such as limited cell sources, cellular injury during collection, demanding cell culture procedures, the invasive nature of local (e.g. intracerebral) cell delivery, immune system and inflammatory responses to allogeneic cells, and the potential difficulties in intergradation of exogenous cells with host brain structures. Mobilization of hematopoietic stem/progenitor cells (HSCs) from the bone marrow has been used clinically in the treatment of hematopoietic diseases and with cancer chemotherapy [5], [6]. In addition to hematopoietic cells, the bone marrow contains mesenchymal stem cells (BMSCs). Both HSCs and BMSCs are multipotent cells that can differentiate into different lineage progenitor cells. Of the three types of stem cells found in the bone marrow, endothelial stem cells (ESCs) represent the majority. They are multipotent and normally give rise to endothelial progenitor cells (EPCs) that can produce vascular endothelial cells (ECs), essential for the formation of blood vessels in angiogenesis. ESCs and EPCs reside in specialized stem cell niches in the adult bone marrow [7]. They express BMSC markers such as CD34 and the endothelial cell marker fetal liver kinase-1 (Flk-1). Transplantation of exogenous ESCs/EPCs has shown great potential in the treatment of ischemic stroke [8], [9], and bone marrow cells can be mobilized into the circulation as a stress-induced protective response after injury under the influence of several chemotactic factors, especially stromal cell-derived factor 1 (SDF-1) [10]–[12]. Mobilized cells can migrate to the ischemic tissue and play a role in recovery and repair by secreting various trophic/growth factors and replace dead cells [3], [13]–[16]. Angiogenesis plays a key regulatory role in maintaining the homeostasis and repair of the nervous system and in neurogenesis [17], [18]. The present investigation focuses on bone marrow derived ESCs/EPCs because of their important roles in angiogenesis and other regenerative potentials.

Several mobilizing cytokines/agents such as granulocyte-colony stimulating factor (G-CSF), paclitaxel, etoposide and parathyroid hormone (PTH) are able to release bone marrow stem cells and/or progenitors into the circulation [5], [6]. G-CSF is currently FDA approved for the mobilization of BMSCs [6]. In an earlier study in 2005, Adams et al., showed that long-term treatment of PTH in mice followed by G-CSF administration could facilitate mobilization of lin^−^/Sca-1+/c/kit+ cells into peripheral blood [19]. In addition, PTH treatment preserves the function of hematopoietic stem cells during multiple rounds of chemotherapy. In a more recent investigation, Brunner et al. compared the mobilization of hematopoietic progenitor cells induced by PTH and G-CSF alone or in combination [20]. In healthy mice, PTH treatment (80 µg/kg for 7 or 14 days) induced increases of hematopoietic progenitor cells in peripheral blood by 1.5 to 9.5 fold. This effect may partly be mediated by the release of endogenous G-CSF since blocking endogenous G-CSF with an antibody significantly reduced the number of mobilized cells after PTH treatment. Interestingly, combination of PTH plus G-CSF showed only a slight additional effect compared to PTH or G-CSF alone. In contrast to G-CSF, PTH does not result in depletion of the bone marrow, which is clinically important for chronic therapy. The authors suggested that “the novel function of PTH on mobilization and regeneration of BMCs may pave the way for new therapeutic options in bone marrow and stem cells transplantation as well as in the field of ischemic disorders” [20]. Based on this available information, we decided to test PTH as a bone marrow cell mobilizer and its regenerative potential after ischemic stroke.

PTH is a peptide hormone involved in regulating peripheral calcium and phosphate homeostasis. It is secreted from the parathyroid gland in response to hypocalcemia, where it stimulates calcium re-absorption in kidneys and intestines, as well as bone re-absorption with the ultimate aim of maintaining proper serum calcium levels. PTH enhances osteogenesis and bone healing in cases of bone fractures and has been used for the treatment of osteoporosis. PTH acts on PTH1 and PTH2 receptors. Activation of the PTH1 receptor in kidney and bone by circulating PTH plays a major and crucial role in regulating Ca^2+^ homeostasis [21]. The PTH2 receptor is synthesized at its highest levels in the central nervous system (CNS), such as in the hypothalamus [22]. Ironically, PTH is present at a very low level in the brain [23]. Since the low concentration of PTH normally does not pass through the blood brain barrier (BBB) [23], [24], the expression of the PTH2 receptor in the brain eventually led to the discovery of the natural ligand for the PTH2 receptor: a 39-residue peptide named tuberoinfundibular peptide (TIP39) [25]. It was noticed that patients with primary hyperparathyroidism show increased numbers of circulating CD34+ EPCs in the peripheral blood concomitant with an increased PTH circulating levels [26], [27], supporting the proportion that PTH can mobilize bone marrow cells into the blood circulation. In a few cardiovascular investigations, PTH has been applied to mobilize stem cells from the bone marrow, and this strategy of by-passing the transplantation procedure has shown improved cardiac repair after myocardial infarction [27]–[30]. At present, there is no report on whether PTH-mobilized bone marrow cells may have beneficial effects after cerebral ischemia.

## Materials and Methods

### Transient focal ischemia animal model

All experimental and surgical procedures were approved by the Institutional Animal Care and Use Committee (IACUC) at Emory University. C57BL/6 (24–28 g, 10–12 weeks old) mice were housed at room temperature with a 12 hr light/dark cycle in the pathogen-free Laboratory Animal Center for Research at Emory University. Occlusions of distal branches of the middle cerebral artery (MCA) were performed according to previous procedures with minor modifications [3], [31]. In brief, 8–10 week old C57BL6 mice were anesthetized by 4% chloral hydrate (100 mg/kg, i.p.). Chloral hydrate has been widely used as a sedation agent in human and animal surgeries/procedures for a number of years. In several systematic examinations and comparisons of different injectable (e.g. ketamine, chloral hydrate, propofol, and pentobarbital) and volatile//inhalable (e.g. isoflurane, sevoflurane and halothane) anesthetics, all tested drugs show side effects and have advantages and disadvantages in a specific situation or specific case [32], [33]. For example, signs of respiratory depression (reduced blood pH, pO_2_, and increased pCO_2_) are commonly seen with injectable anesthetics, while metabolic disturbances such as significantly increased lactate concentration are observed with inhalable anesthetics [32], [33]. Moreover, marked increases of blood glucose concentration occurs under both types of anesthetics [32]. Significantly in stroke investigations, NMDA receptor antagonism shared by many anesthetics may be neuroprotective and thus a confounding factor interfering with data analysis. Chloral hydrate was chosen in this investigation due to its stable and well controlled anesthetic action and negligible effect on NMDA receptors [34].

The right MCA branches were permanently ligated using a 10-0 suture (Surgical Specialties Co., Reading, PA) accompanied by a bilateral 7-min ligation of the common carotid arteries (CCA). During CCA occlusion, barrel cortex blood flow was reduced to less than 20% as measured by laser Doppler scanning. Body temperature was monitored during surgery and maintained at 37.0°C using a temperature control unit and heating pads. The mortality rate due to ischemic surgery and/or anesthesia failure was around 7% in these experiments. Animals were euthanized and decapitated at different time points after ischemic stroke. Brains were immediately removed, mounted in optimal cutting temperature compound (Sakura Finetek USA, Inc., Torrance, CA) and stored at −80°C for further processing.

### Experimental groups and drug administration

Three days after behavioral training, mice were randomly divided into ischemic stroke-saline control group and ischemic stroke-PTH treatment group. In the stroke-PTH group, PTH 1-34 (80 µg/kg; BACHEM Americas INC, Torrance, CA) was given by intraperitoneal (i.p.) injection one hour after stroke induction and daily for 6 constitutive days. Control animals received saline i.p. injections at the same time points. To label proliferating cells, 5-bromo-2-deoxyuridine (BrdU) (Sigma) was administered to all animals (50 mg/kg/day, i.p.) beginning on day 3 after stroke induction and once daily until sacrifice at 6, 14 and 21 days after ischemia.

### Flow cytometry

Six days after stroke induction, 1 ml of peripheral blood was collected from each mouse by aspiration from the left heart ventricle. Mononuclear cells were separated by density-gradient centrifugation using 1.077 g/ml Histopaque solution (GE Healthcare Biosciences, PA, USA), purified, resuspended in phosphate buffered saline containing 0.5% bovine serum albumin, and then incubated with the following antibodies: FITC-conjugated CD34, PE-conjugated VEGFR2/Flk1/KDR (BD Biosciences, San Jose, CA, USA). Labeled cell populations were measured by FACSCalibur (BD Biosciences). FACS (Fluorescence Activated Cell Sorting) data was analyzed by FlowJo 7.0 software (Ashland, OR, USA). FACS analysis was performed using a variety of controls including unstained samples, isotype antibodies, and single-stained samples for determining appropriate gates, voltages, and compensations required in multivariate flow cytometry.

### Western blot analysis

Fresh brain tissue was isolated from the peri-infarct area, defined as the region within 500 µm from the edge of the infarct area. Western blot analysis was performed to analyze protein expression in the penumbra following previous procedures [35]. In brief, brain tissue was lysed in lysis buffer containing 25 mM Tris–HCl pH 7.4, 150 mM NaCl, 5 mM EDTA, 0.1% SDS, 2 mM sodium orthovanadate, 100 mM NaF, 1% triton, leupeptin, aprotinin, and pepstatin with continuous manual homogenization. After 30 min, the lysate was spun at 17,000 rpm for 15 min at 4°C and the supernatant was collected. The protein concentration was determined using the Bicinchoninic Acid (BCA) protein assay (Pierce, Rockford, IL). Equal amounts of protein (50 µg) were resolved on SDS-PAGE using gradient gels (6–20%) and gels were blotted onto PVDF membranes (Amersham, Buckinghamshire, UK), blocked with 5% BSA in TBST buffer (20 mM Tris, 137 mM NaCl and 0.1% Tween) and incubated overnight with primary antibodies against SDF-1 (1∶200, Cell Signaling), EPOR (1∶500, Santa Cruz), vascular endothelial growth factor (VEGF) (1∶500, Santa Cruz), brain-derived neurotrophic factor (BDNF) (1∶500, Santa Cruz), Tie-1 (1∶200, Santa Cruz), Angiopoietin-1 (Ang-1) (1∶500, Santa Cruz), and Flk-1 (1∶500, Santa Cruz). After 3 washes with TBST, blots were incubated with alkaline phosphatase-conjugated anti-mouse or anti-rabbit IgG antibodies (Promega, Madison, WI) for 2 hrs at room temperature. Finally, membranes were washed with TBST and the signal was developed by the addition of 5-bromo-4-chloro-3-indolylphosphate/nitroblue tetrazolium (BCIP/NBT) solution (Sigma), quantified, and analyzed by the imaging software ImageJ (NIH, Bethesda, MD, USA). The level of protein expression was normalized to β-actin (1∶2500, Sigma) controls.

### TTC staining of infarct volume measurement

Three days after sham or MCA occlusion surgery, animals in different groups were sacrificed for assessment of brain infarct formation. 2,3,5-triphenyltetrazolium chloride (TTC) staining was used to reveal damaged/dead brain tissue. Brains were removed and placed in a brain matrix then sliced into 1-mm coronal sections. Slices were incubated in 2% TTC (Sigma, St. Louis, MO) solution at 37°C for 5 min, then stored in 10% buffered formalin for 24 hrs. Digital images of the caudal aspect of each slice were obtained by a flatbed scanner. Infarct, ipsilateral hemisphere, and contralateral hemisphere areas were measured using ImageJ software (NIH, Bethasda, MD). Infarct volume was calculated using the indirect method [36].

### Immunohistochemical assessment and cell counting

Preparation of brain sections was performed as previously described [37]. In brief, coronal brain sections were cut at 10 µm thickness using a cryostat (Ultrapro 5000; St. Louis, MO). Sections were dried on a slide warmer for 30 min, fixed with 10% buffered formalin for 10 min, washed with −20°C pre-cooled ethanol∶acetic acid (2∶1) solution for 10 min and then permeabilized with 0.2% Triton-X 100 (in PBS) for 5 min. Sections were then blocked with 1% fish gel (Sigma) in PBS for 1 hr at room temperature, and incubated with the primary antibodies Glut-1 (1∶800; Milipore, MA, USA) and NeuN (1∶400; Millipore, MA, USA) overnight at 4°C. Slides were incubated with anti-mouse and anti-rabbit secondary antibodies for 2 hrs at room temperature. Vectashield mounting media for fluorescence (Vector Laboratory, Burlingame, CA) or ProLong AntiFade (Invitrogen, Grand Island, NY) were used to cover-slip slides in preparation for microscopy and image analysis. Fluorescence was visualized by fluorescence microscopy (BX51; Olympus, Tokyo, Japan). For BrdU (1∶600, AbD serotec) staining, slides were incubated in 2N HCl at 37°C for 40 min, followed by 0.2% Trition-X 100 incubation for 40 min. For DCX (1∶50, Santa Cruz Biolechnology) staining, animals were transcardially perfused with warm saline and buffered 10% formalin (VWR, PA, USA).

Cell counting was performed following the principles of design-based stereology. Systematic random sampling was employed to ensure accurate and non-redundant cell counting [38]. Every section under analysis was at least 100 µm away from the next to ensure that the same cells were not counted twice. For each animal, six 10-µm thick sections spanning the entire region of interest were randomly selected for cell counting. Counting was performed on 6 randomly selected non over-lapping fields per section. Sections from different animals represent the same area in the anterior-posterior direction. Vessel density was measured using ImageJ (NIH). Distance and quantification of neuroblast migration was performed as described before [39]. Briefly, proliferating cells in the SVZ and their migration toward the ischemic cortex were captured in a series of 6–10 images, depending on the distance of migration, at 10× or 20× magnification with eight sections analyzed per animal. Analyses were performed on coronal brain section images taken within the region of interest including the SVZ migration tract and the peri-infarct region. Cells co-labeled with BrdU and DCX were counted as newly formed neural progenitors. Migration distance (µm) was evaluated from the SVZ to the furthest co-labeled DCX and BrdU-positive cell, determined by Z-stack imaging. Cell counting was performed by an individual who was blind to the experimental groups.

### Local Cerebral Blood Flow Measurement (LCBF)

Laser Doppler scanner imaging of cortical cerebral blood flow was conducted as previously described [40] at three time points: immediately before MCA ligation, during the 7 min bilateral common carotid artery ligation, and 14 days after ischemia. Briefly, animals were anesthetized with an injection of 4% chloral hydrate solution and an incision was made to expose the skull above the territory of the right MCA. The laser was centered over the right coronal suture. Different from the conventional Laser Doppler probe that measures a small point of blood flow, the scanner method measures a 2.4×2.4 mm square area using the laser Doppler perfusion imaging system (PeriFlux System 5000 - PF5010 LDPM unit, Perimed, Stockholm, Sweden). This scanning measurement largely avoids inaccurate or bias results caused by inconsistent location of the laser prob. Data was analyzed using the LDPI Win 2 software (Perimed AB, Stockholm, Sweden).

### Evaluation of neurological function deficits: adhesive removal test

The adhesive removal test is a sensitive method to assess sensorimotor deficits in focal cerebral ischemic mice [41]. In brief, a piece of adhesive tape was placed on each (right and left) forepaw and the time-to-contact (latency) and the time-to-remove (removal) the tape was recorded. Animals were trained for three days (1–2 trials per day) before stroke induction until the mice could take the adhesive tape off their paws within 12 sec to get a basal level of performance. Animals were tested before stroke and 6, 14 and 21 days after ischemia by an investigator who was blind to the experimental groups. The mean time (in seconds, average of 5 trials) required to detect (time-to-contact) and remove (time-to-remove) the adhesive tape from the left forepaw was recorded. All testing trials were conducted during the day.

### Statistical analysis

All results are expressed as mean ±S.E.M. Statistical comparisons, using Graph Pad Prism 5 (Graph Pad Software, Inc., San Diego, CA), were made with Student's *t-test* or two-way analysis of variance (ANOVA) with Bonferroni's *post-hoc* analysis to identify significant differences. *P*<0.05 was considered significant for all comparisons.

## Results

### Mobilization of CD34+/Flk1+ endothelial progenitor cells after ischemic stroke and PTH treatment

**Figure 1:**
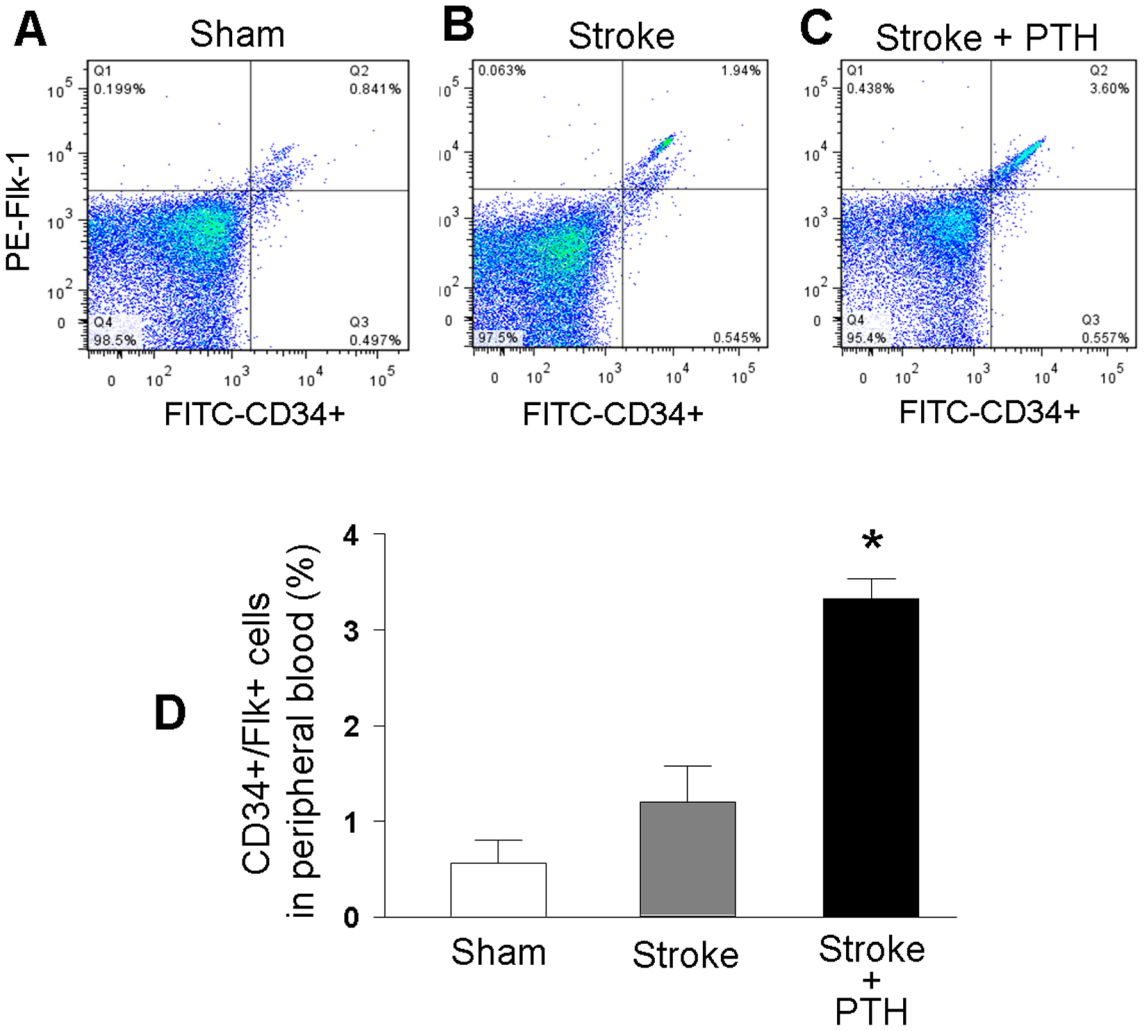
PTH increased the endothelial progenitor cells in the peripheral blood. Six days after stroke with PTH or saline treatment, blood samples were collected. FITC-conjugated CD34 and PE-conjugated VEGFR2/Flk1/KDR labeled cell populations were measured by BD FACSCalibur flow cytometer. **A**–**C**. Representative results of Fluorescence Activated Cell Sorting (FACS) are shown in A (sham control), B (stroke plus saline), and C (stroke plus PTH). **D**. The bar graph shows quantified data with a significant increase in CD34+/Flk1+ cells in stroke plus PTH group. N = 5 animals in each of the three groups; * *P*<0.05 vs. sham and stroke controls.

Adult mice were subjected to a focal ischemic insult targeting the barrel cortex [42], [43]. PTH 1-34 (80 µg/kg, i.p.) and saline control were injected daily starting from 1 hr after stroke and continued once a day for 3–6 days. This dosage was selected based on previous studies showing that this dosage of PTH mobilized CD34+ cells and increased homing to the ischemic heart [20], [44], [45]. At day 6 after the treatment and stroke, blood samples were collected and examined using flow cytometry. CD34 is a typical BMSC marker and Flk-1 is a cell surface receptor protein that is commonly used as a marker for ESCs and EPCs [46]. We focused on CD34 and Flk1 positive (CD34+/Flk+) cells that are well characterized as bone marrow derived ESCs/EPCs. In peripheral blood samples from control and stroke groups, CD34+/Flk1+ cells in stroke animals showed a tendency to increase but were not statistically different from that in sham control animals (n = 5 animals in sham and stroke group, respectively) (Fig. 1). Stroke mice that received PTH treatment showed a marked increase, i.e. more than double the amount in controls, of CD34+/Flk1+ cells in peripheral blood (*P*<0.05) (Fig. 1).

### Systemic injection of PTH did not show significant effect on ischemia induced infarct formation

It was reported that PTH related peptides protected against kainic acid excitotoxicity in cerebellar granule cell cultures [47]. It has not been tested whether PTH is neuroprotective in vivo after ischemic stroke. To understand whether PTH may show a direct neuroprotective effect when administrated via a systemic injection, we measured infarct formation 3 days after the onset of MCA occlusion in mice that received saline (n = 9) and PTH treatment (80 µg/kg, i.p. 1 hr after stroke and ×3 days, n = 10). This peripheral treatment of PTH showed no protective effect against ischemia-induced infarct formation (Fig. S1), which is consistent with the previous reports that systemic administration of PTH can not pass through the blood brain barrier [23], [24], even though PTH may show a protective effect in vitro.

### Expression of neurovascular regenerative factors in the ischemic cortex

**Figure 2:**
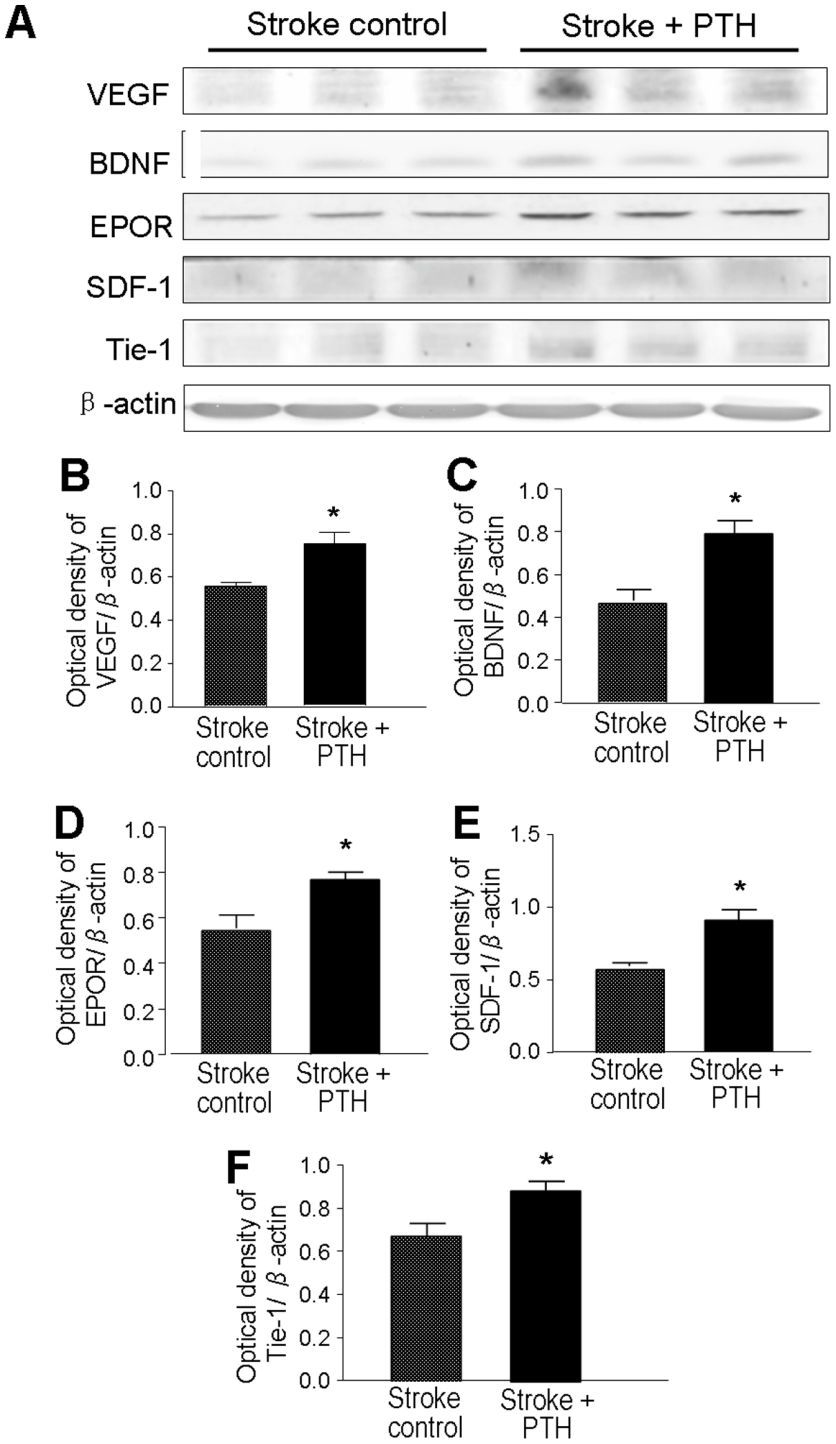
Effects of PTH on the expression of angiogenic and neurotrophic factors. The expression of key pro-regenerative factors in the peri-infarct region was detected using Western blot analysis. **A**. Electrophoresis gels show the protein levels of VEGF, BDNF, EPOR, SDF-1, and Tie-1 in the ischemic peri-infarct region in different animals 6 days after stroke. **B** to **F**. Densitometry analysis of the expression of BDNF (B), VEGF (C), EPOR (D), SDF-1 (E), and Tie-1 (F). Gray intensity was normalized against β-actin and quantified. PTH treatment significantly enhanced the expression of all these factors shown in A, compared with the stroke-saline group. N = 3 animals for each test; * *P*<0.05.

Six days after stroke and PTH or saline treatment, expression of regenerative factors VEGF, EPO receptor (EPOR), Ang-1, Tie-1, Flk-1, BDNF and SDF-1 in the ischemic peri-infarct region were analyzed using Western blotting. Compared with stroke-vehicle control mice, the levels of pro-angiogenic factors VEGF, EPOR, Tie-1 and the neurotrophin BDNF significantly increased in PTH treated mice (n = 3 in stroke and stroke plus PTH groups, respectively; *P*<0.05) (Fig. 2). The expression of Ang-1 and Flk-1 showed a trend of increasing but didn't reach statistical difference (data not shown). There was, however, a significant increase in the expression of migration chemoattractant SDF-1 in the PTH treatment group compared with the stroke control group (Fig. 2).

### PTH treatment increased angiogenesis and local blood flow restoration at the peri-infarct region after focal cerebral ischemia

**Figure 3:**
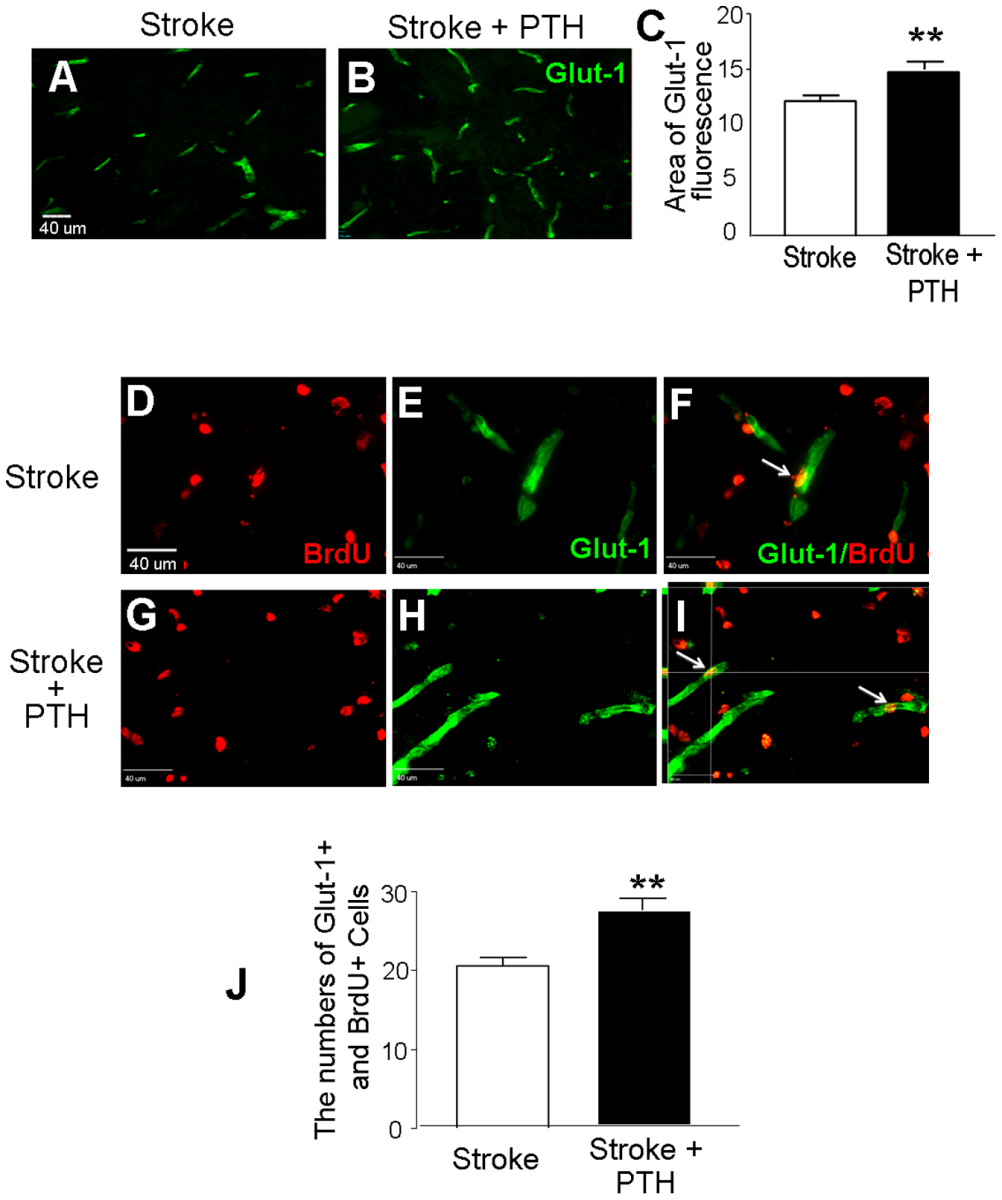
PTH enhanced angiogenesis at the peri-infarct region 14 days after stroke. Fourteen days after stroke, endothelial cells and cell proliferation at the peri-infarct region was inspected using immunohistochemical methods. **A** and **B**. Representative Glut-1 (green) staining of vascular endothelial cells in the brain section from stroke/saline and stroke/PTH mice. **C**. The area of Glut-1 fluorescent reactivity in stroke/saline and stroke/PTH groups. **D** to **I**. Fluorescent labeling of Glut-1+ (green) and BrdU+ (red) cells. F and I are 3-D images showing co-localized labeling of Glut-1 and BrdU, indicative of angiogenesis. **J**. Quantified data of fluorescent reactivity of Glut-1/BrdU double positive cells; PTH treatment significantly promoted angiogenesis. N = 6; ** *P*<0.01 vs. stroke control.

Since CD34+/Flk1+ ESCs/EPCs increased in the circulation and pro-angiogenic factors were elevated in the brain by PTH, we tested the hypothesis that post-ischemia angiogenesis could be enhanced after chronic PTH treatment. Glut-1 is a specific marker for glucose transporter in brain vascular endothelial cells. PTH treatment increased the Glut-1+ vessel staining 14 days after stroke (n = 6 in stroke and the stroke plus PTH group, respectively; *P*<0.05) (Fig. 3A–3C). We then counted the number of Glut-1+ microvessels co-labeled with BrdU in the peri-infarct region 14 days after stroke. Compared with the stroke-saline group, the mice receiving PTH treatment showed significantly more Glut-1+/BrdU+ microvessels, suggesting increased formation of new vessels or angiogenesis in the post-stroke brain (Fig. 3D–3J).

**Figure 4:**
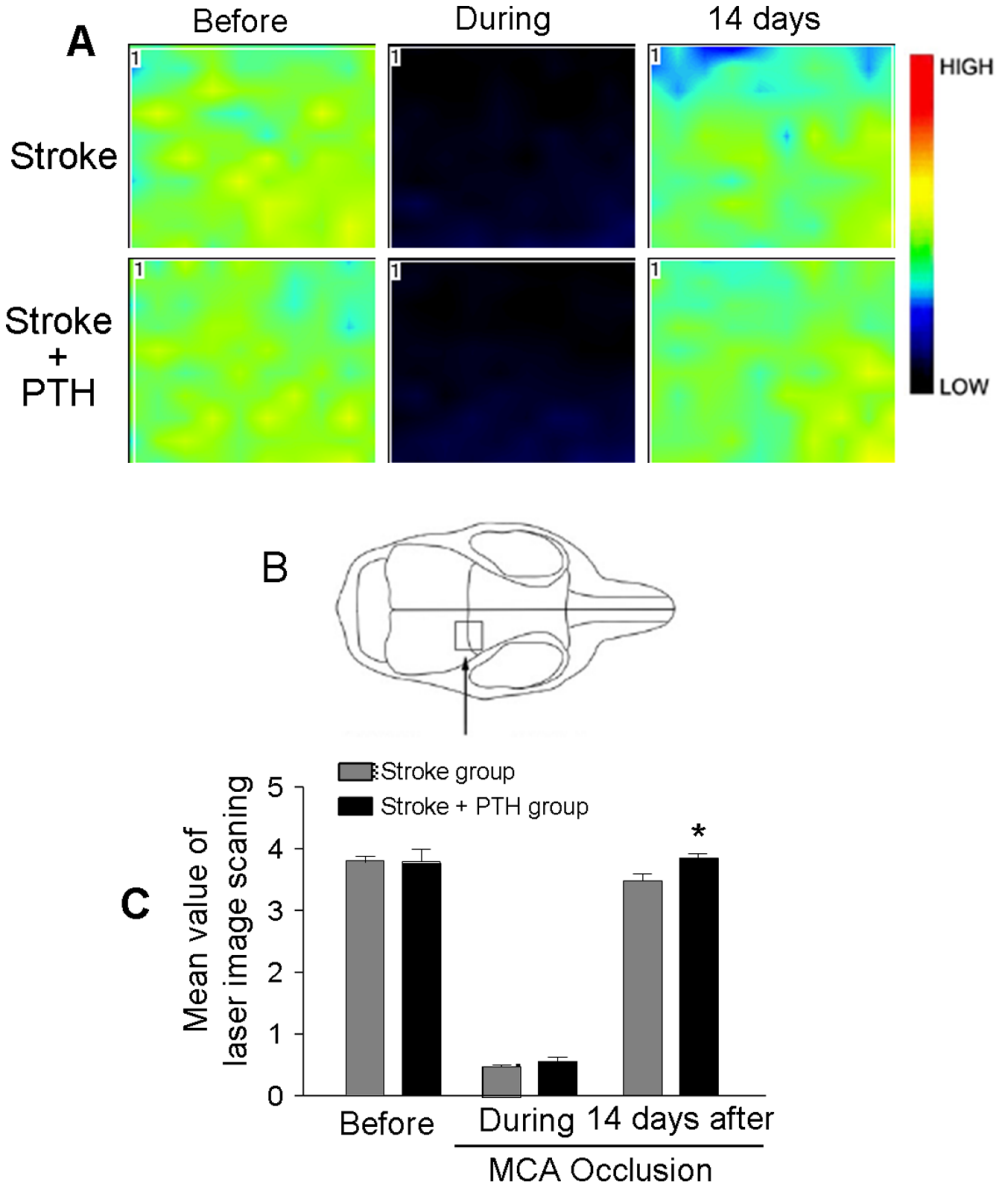
Recovery of local cerebral blood flow in the ischemic cortex. Local cerebral blood flow was measured using the PeriScans laser image scanner 14 days after stroke. **A**. Laser scanning images of LCBF in stroke/saline mice and stroke/PTH treatment mice before, during and 14 days after MCA occlusion. B. Sketch of the mouse skull showing the imaging area (square frame with arrow) for LCBF. The total scan area was 5.76 mm^2^. **C**. Quantification of LCBF measurements using the laser Doppler scanner. In this focal ischemia model of barrel cortex stroke, LCBF could largely recover 14 days after stroke. A greater recovery was seen with PTH treatment. N = 8 animals in each group; * *P*<0.05 vs. stroke control.

Local blood flow recovery is needed for sustained cell survival and tissue repair after stroke. To demonstrate that the PTH-promoted angiogenesis could result in functional vasculatures, we measured the local cerebral blood flow using a Laser Doppler Scanner at 14 days after stroke. The scanning imaging showed that stroke mice that received PTH had significantly greater recovery of local blood flow compared with the stroke-saline group (n = 8 in stroke and the stroke plus PTH group; *P*<0.05) (Fig. 4).

### PTH enhanced neuroblast migration and neurogenesis after focal ischemia

**Figure 5:**
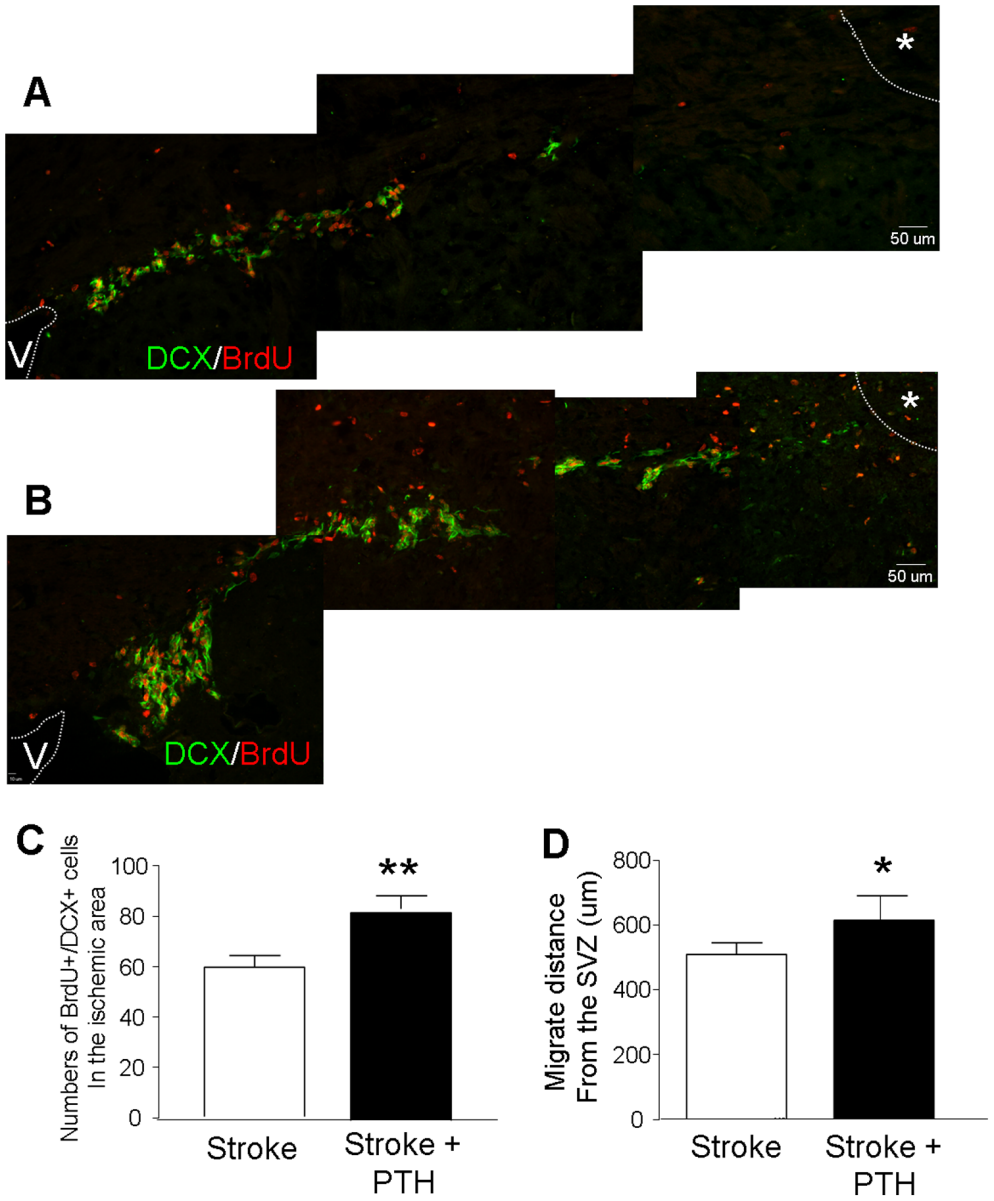
PTH treatment enhanced neuroblast migration from the SVZ to the ischemic cortex. The neuroblast migration and regenerative activity along the ipsilateral SVZ to ischemic cortex migration route was examined by immunostaining of migrating neural progenitor marker DCX (green) and the proliferating marker BrdU (red) 6 days after the onset of ischemia. **A**. In the post ischemic brain there was significant neuroblast migration from the SVZ to the ischemic barrel cortex (*). Noticeably more DCX and BudU positive cells can be seen in the migration path of PTH treated animals (B) than in the stroke/saline brain (A). **C**. The total number of DCX+/BrdU+ cells in six sections per animal was counted under a fluorescent microscope. Double-labeling of DCX/BrdU in white matter of ischemic stroke-saline (C) and PTH treated mice (D) 6 days after ischemia. PTH treatment promoted more DCX/BrdU-positive cells moving toward the ischemic cortex (F). The number of DCX+/BrdU+ cells in stroke control and stroke/PTH groups. PTH treatment markedly augmented the proliferating activity among DCX+ cells. **D**. PTH treatment increased the average migration distance from the SVZ toward the ischemic barrel cortex. N = 6 in stroke/saline group and 7 in stroke/PTH group; * *P*<0.05 vs. stroke controls, ** *P*<0.01 vs. stroke controls.

Cerebral ischemia stimulates SVZ-derived neural progenitor cells or neuroblasts to migrate toward the ischemic cortex [48]. The ischemic insult increases expression of SDF-1 in the ischemic and peri-infarct region, which plays a major chemoattractive role in this process [48]. Since PTH mobilized CD34+/Flk1+ cells express high levels of SDF-1, an enhanced directed migration of neuroblasts was predicted in the event the mobilized cells could home to the ischemic cortex. Migrating neuroblasts were identified by expression of the microtubule associated protein doublecortin (DCX) [49]. Immunofluorescence double labeling of DCX and the proliferation marker BrdU was performed at 6 days after stroke. At this time, DCX+/BrdU+ cells could be observed in the white matter area between ipsilateral SVZ and ischemic cortex (Fig. 5), while these cells were not detectable in non-stroke controls (data not shown). The number of DCX+/BrdU+ cells was significantly greater in stroke mice that received PTH treatment (n = 7) than in stroke-saline mice (n = 6) (Fig. 5). Moreover, in the PTH-treated ischemic brain, the neuroblast migration distance from SVZ was also significantly longer compared with that in stroke-saline group (Fig. 5).

**Figure 6:**
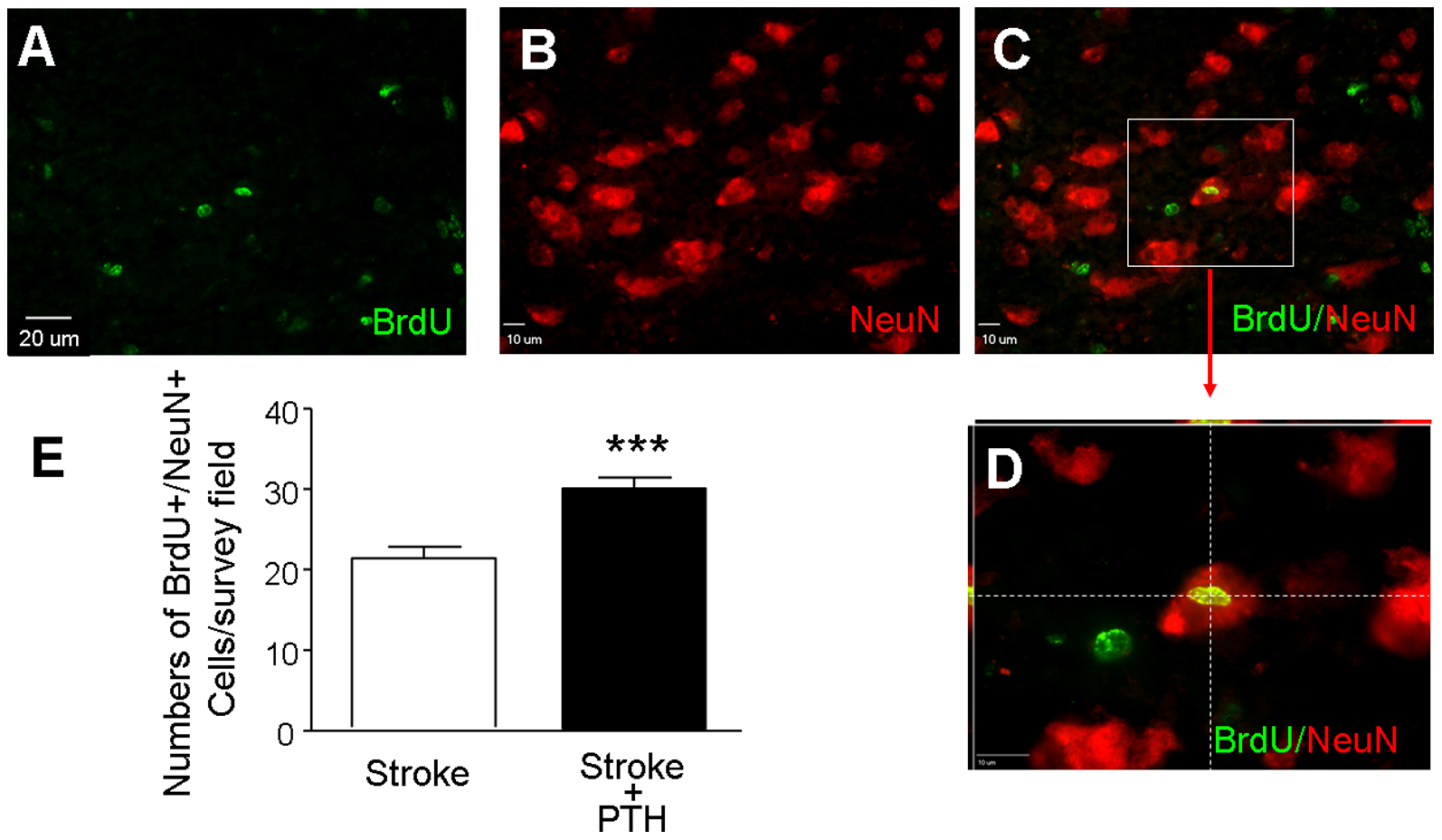
PTH enhanced neurogenesis at the peri-infarct region 21 days after stoke. Neurogenesis in the peri-infarct region was examined by the colocalization of the neuronal marker NeuN (red) and the proliferation marker BrdU (green) 21 days after stroke. **A** to **C**. Immunostaining images of NeuN+ cells (A), BrdU+ cells (B) and the merged image of NeuN+ and BrdU+ cells (C). **D**. Enlarged images of colocalization of NeuN/BrdU from the frame shown in C. **E**. Cell counts were performed in six randomly chosen fields per section in the peri-infarct region. The total number of cells in six sections was summarized for each mouse. Cell counts show increased numbers of Neu+/BrdU+ cells in the PTH treatment group compared with the stroke/saline group. N = 6 animals each group, *** *P*<0.001 vs. stroke controls.

To determine whether migrated neuroblasts differentiated into neurons in the peri-infarct region where they are needed for tissue repair, cells were co-stained with NeuN and BrdU 21 days after stroke. In stroke mice that received PTH, there was a much greater number of NeuN+/BrdU+ cells compared with the stroke controls (n = 6 in stroke and stroke plus PTH group, respectively; *P*<0.05) (Fig. 6).

### PTH treatment attenuated ischemia induced neurological deficits

**Figure 7:**
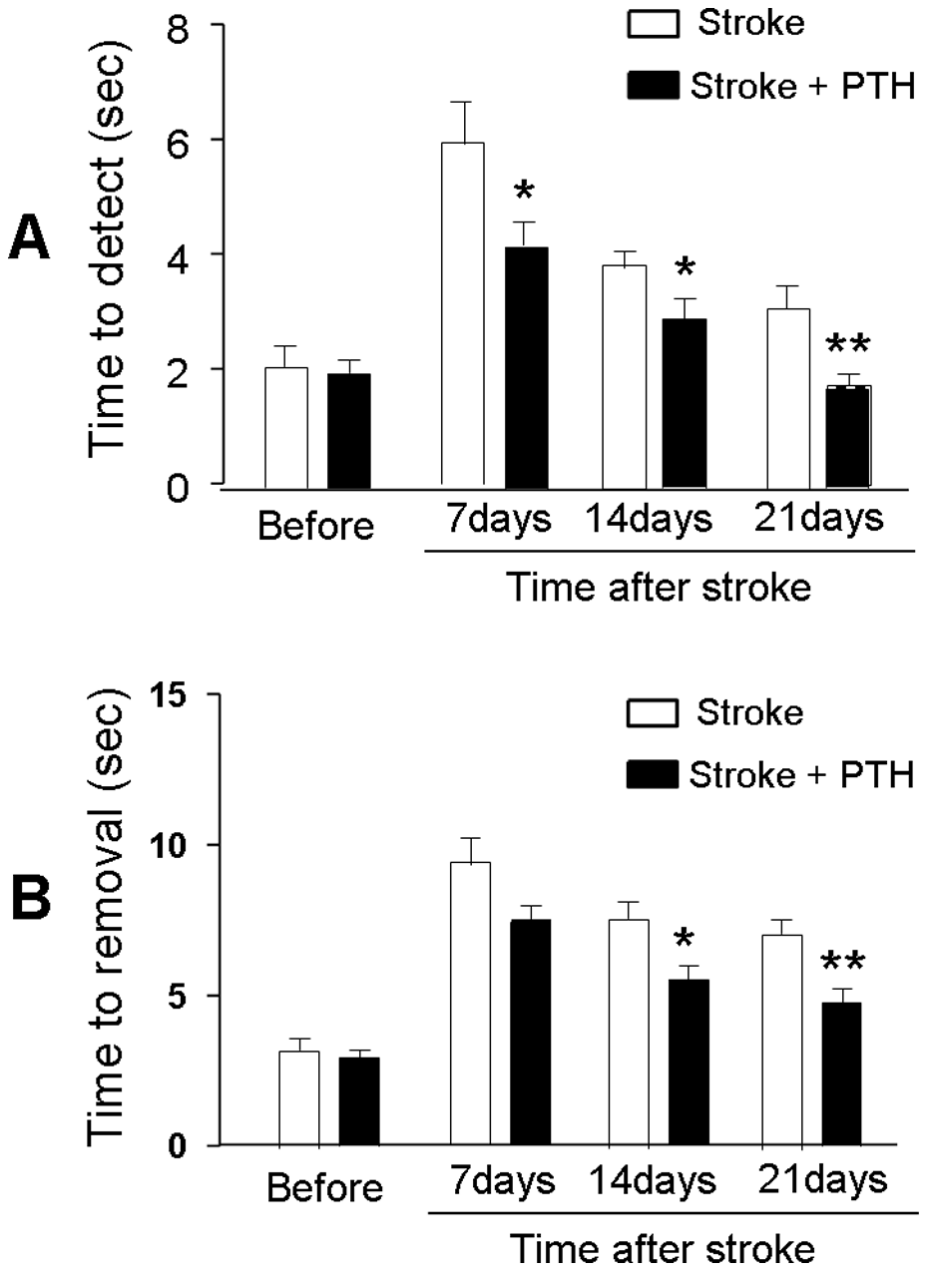
PTH treatment promoted functional recovery after stroke. The adhesive-removal test was performed to evaluate sensorimotor deficits of the mice before, and 7 to 21 days after focal cerebral ischemia. **A**. All mice showed impaired function in this test (increased time to detect) after stroke. Mice that received PTH, however, exhibited significantly improved sensorimotor activity (faster detection of the sticky dot) at all time points tested. **B**. The time to removal of the sticky dot from the affected left paw was significantly increased in all stroke animals at 7 days after stroke. PTH treatment, however, improved this sensorimotor activity so that the time to removal was significantly shorter 14 days after stroke. N = 11–20 animals, * *P*<0.05, ** *P*<0.01 vs. stroke controls at the same time point.

The distal MCA branch occlusion model targets the barrel somatosensory field that controls whisker and fore-limb function [50]–[52]. The adhesive removal task is an established sensitive measure of sensorimotor function in mouse focal ischemic stroke [41], [53]. The test was performed 6 to 21 days after the focal cerebral ischemia. The time needed for stroke animals to feel (time to detect) and remove (time-to-remove) the sticky dot from the left paw was markedly prolonged after stroke due to the ischemic damage in the right sensorimotor cortex [54]. This functional impairment was significantly attenuated in mice receiving PTH treatment compared with vehicle-treated mice. In PTH-treated mice, the time-to-detect and the time to remove were both significantly improved compared to stroke-saline controls (n = 11–20 in stroke and stroke plus PTH groups at different time points; *P*<0.05) (Fig. 7).

## Discussion

In this study, we examined the effects of PTH administration on bone marrow stem cell mobilization and its effects on regenerative mechanisms after focal cerebral ischemic stroke. Using flow cytometry and double labeling with CD34 and Flk-1, we confirmed a mobilized release of bone marrow ESCs/EPCs into the peripheral blood after 6-day PTH treatment in stroke mice. Immunohistochemical examination using the proliferation marker BrdU and specific cell type markers demonstrates that PTH treatment enhances angiogenesis, neuroblast migration and neurogenesis in the post-ischemic brain. Apart from many previous studies, no harvest and transplantation of bone marrow cells was performed in this investigation. We suggest that the PTH-mobilized ESCs/EPCs from the bone marrow and the functional benefits represent a new potential regenerative therapy for tissue repair after ischemic stroke.

As an endogenous hormone, PTH acts to increase the concentration of Ca^2+^ in the blood, whereas calcitonin (a hormone produced by the parafollicular cells of the thyroid gland) decreases Ca^2+^ concentration. It is a concern that chronic use of PTH may lead to hypercalcemia. The dosage (80 µg/kg/day) used in this investigation has been widely tested in previous investigations in animals models [20], [44], [45]. In a clinical assessment of postmenopausal women, 40 µg/kg daily PTH 1-34 (i.p.) for 21 months caused hypercalcemia in 28% of the subjects [55]. Although one cannot directly compare a drug dosage for humans to the dosage in animals or vise versa, it is possible that the PTH dosage of 80 µg/kg may cause hypercalcemia in some patients. However, PTH was injected only for 6 days for the treatment of stroke, so the hypercalcemia effect should be minimal in this relatively short period of PTH administration. PTH has a relatively short half-life (approximately 4 min) [56]. Thus, the hypercalcemic effect or other side effects of the PTH therapy demonstrated in stroke animals is likely to be mild and transient. Even so, because of the negative consequences of hypercalcemia such as kidney failure, hypertension, irregular heartbeat, and dehydration, cautions should be taken to recognize any side effect and risk factor for cardiovascular and cerebrovascular events. In more translational investigations, these potentially negative impacts should be valuated in animal models and clinical trials. Interestingly, a clinical survey on 651 stroke patients using *Bivariate* analysis showed that the patients with the highest Ca^2+^ levels measured 72–96 hrs after stroke were associated with less stroke severity and better 3-month functional and independence scale outcomes [57]. This was perhaps due to the possibility that the higher blood Ca^2+^ is an indication of lower intracellular Ca^2+^ concentration that helps to lessen excitotoxic cell death.

In rat models of focal cerebral ischemia, administration of CD34+ EPCs improved functional outcomes [8], [58], and was attributed to tissue regeneration promoted by angiogenesis [16], [24]. In humans, circulating levels of EPCs tend to reflect the temporal profile of recovery from 7 to 14 days after stroke onset [59], [60]. Using female mice that previously underwent sex-mismatched bone marrow transplantation from enhanced green fluorescent protein (eGFP) expressing mice, it was shown that G-CSF plus Stem cell factor (SCF) could stimulate release of EPCs from the bone marrow and these cells have the ability to home to the injured brain area, differentiate into ECs and increase vessel formation after permanent middle cerebral artery occlusion [61]. These observations support the idea that ESCs/EPCs mobilized from the bone marrow can contribute to brain tissue repair via homing to the ischemic brain. Our data support that endogenously released CD34+ ESCs/EPCs by PTH can promote functional recovery via the cellular mechanism of increased angiogenesis. Several trophic factors participate in the angiogenic response after ischemic stroke such as VEGF/VEGF-R, Ang-1/Ang-2, Tie-1 and Tie-2 receptors [62], [63]. In addition, SDF-1/CXCR4 signaling plays an important role in angiogenesis by increasing expression of VEGF through the activation of the PI3K/Akt pathway [64]. Many of these factors are up-regulated in the stroke mice that received PTH. Recent research reveals that post-stroke angiogenesis is tightly coupled to neurogenesis and possibly mediated by the release of SDF-1, Ang-1 and BDNF from proliferating endothelial cells [65], [66]. We suggest that although the observed angiogenic activity and increased migration of neuroblasts to the ischemic cortex is a result of mobilized ESCs/EPCs that home to the ischemic cortex, the increased NeuN/BrdU positive cells in the peri-infarct region most likely come from the migrated neuroblasts that originated in the SVZ.

It has been demonstrated that PTH does not pass through the BBB so the brain PTH level is negligible or very low [23], [24]. Since cerebral ischemia may damage the BBB and increase its permeability, PTH circulating in the blood may leak into the brain tissue. While this possibility exists, since the animal model of barrel cortex stroke in our investigation is a “mini-stroke” model that only affects a small restricted part of the cortex [31], the leakage of PTH in this model should be minimum. The observation that systemic administration of PTH did not show significant effect on infarct volume supports the prediction that PTH may not pass through BBB, and that PTH shows its regenerative effects via its peripheral action of mobilizing bone marrow cells. The increased local cerebral blood flow 14 days after stroke and PTH treatment was mostly likely due to increased regeneration such as angiogenesis during the chronic phase after stroke. In any event, a decisive demonstration of the mechanism by which PTH shows regenerative benefits in the ischemic brain remains to be further explored due to technical limitation in tracking mobilized bone marrow cells.

Although the tracking methods for transplanted exogenous stem cells are available, there are few established methods to directly track migration and homing of endogenously mobilized bone marrow cells. The sex-mismatched eGFP-expressing mice may be the only stroke model that can be more specifically tested for this purpose [61]. This mouse may be of use for a further investigation when it is available. Bone marrow cell markers may provide some information, however they are not specific for these cells. For example, CD34 is a widely used marker in hematopoietic stem cell mobilization in the peripheral blood, however, CD34 is expressed widely on vascular endothelium, including that in the CNS [67], [68], which prevents conclusive identification of the bone marrow origin of these cells. We recognize that more specific experimental evidence is needed to distinguish the PTH direct central effects from its mobilization action in the bone marrow. Nevertheless, if PTH does have a direct action in the brain, our investigation still suggests a new therapy of using PTH in regenerative treatment after ischemic stroke.

In summary, PTH treatment after stroke can mobilize ESCs/EPCs from the bone marrow into peripheral blood circulation. Compared to transplantation of allogeneic and autologous stem cells/progenitor cells, mobilizing bone marrow cells has obvious advantages, such as reduced adverse immune response, a non-invasive nature, and manageable continuous supply of the cells. ESCs/EPCs in the peripheral circulation have the ability to migrate and home to the ischemic region and optimize the microenvironment in the post-stroke brain. PTH can promote angiogenesis, enhance neuroblast migration and neurogenesis in the ischemic cortex, and improve the local blood supply. All these direct and indirect actions ultimately enhance tissue repair and functional recovery after ischemic stroke. As this is the first report on this PTH therapy for ischemic stroke for the demonstration of the efficacy and feasibility, PTH treatment was initiated at 1 hr after stroke followed by repeated administrations for 6 days. We expect that even more delayed treatment of PTH, e.g. several hrs after stroke, can be beneficial in promoting chronic angiogenesis and other tissue repair process. This possibility, however, remains to be further evaluated in a more translational investigation.

## Supporting Information

Figure S1 **Cerebral ischemia-induced infarct volume was not affected by PTH treatment.** Three days after stroke, animals that received saline or PTH treatment (80 µg/kg, i.p. 1 hr after the onset of MCA occlusion and once every day for 3 days) were sacrificed and brain sections were subjected to TTC staining for the measurement of brain infarct volume. The PTH treatment, however, did not affect the ischemia-induced infarct formation. N = 9 in stroke-saline group and 10 in stroke plus PTH group. *P*>0.05 between the two groups.
(TIF)
