# Supplementary figures and images for: Mobilization of Endogenous Bone Marrow Derived Endothelial Progenitor Cells and Therapeutic Potential of Parathyroid Hormone after Ischemic Stroke in Mice
Source: PLoS One. 2014 Feb 4;9(2):e87284. doi: 10.1371/journal.pone.0087284 (PMC3913619; doi:10.1371/journal.pone.0087284)

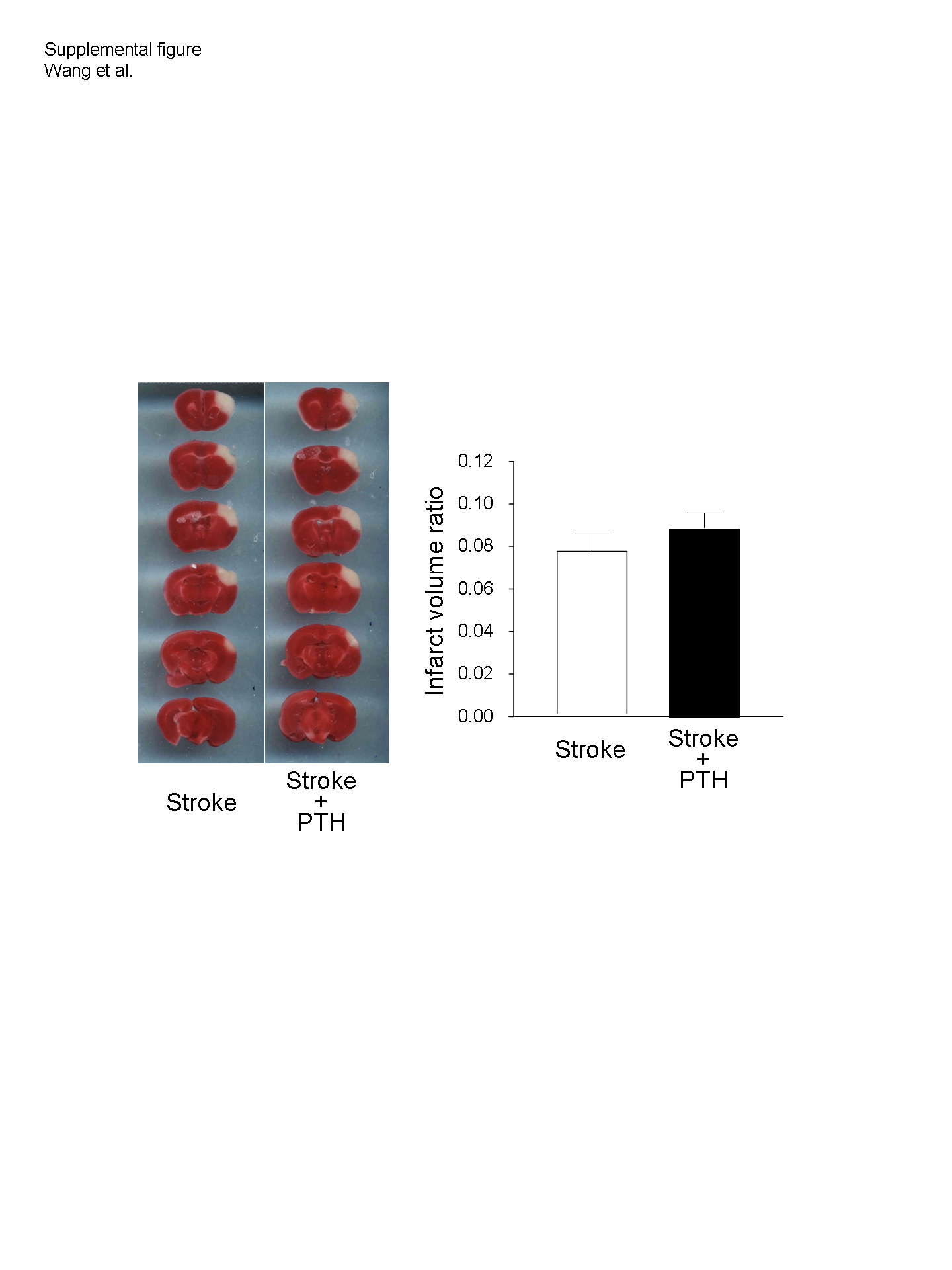

Supplement: Figure S1 — Cerebral ischemia-induced infarct volume was not affected by PTH treatment. Three days after stroke, animals that received saline or PTH treatment (80 µg/kg, i.p. 1 hr after the onset of MCA occlusion and once every day for 3 days) were sacrificed and brain sections were subjected to TTC staining for the measurement of brain infarct volume. The PTH treatment, however, did not affect the ischemia-induced infarct formation. N = 9 in stroke-saline group and 10 in stroke plus PTH group. P>0.05 between the two groups. (TIF) [file pone.0087284.s001.tif]
